# 

**Published:** 1854-07

**Authors:** 


					﻿X,; .ii.j
J U L Y.
[Vol. X.
•<
BUFFALO
MEDICAJ JOURNAL
I	»
AX D
llemew
OF
*
MEDICAL AND SUE 1CAL SCIENCE.
*
■ AUSTIN FLINT, M. I)..	}
SANFORD B. HUNT, AL I)., $ Lnrr0BS-
- '
BUFFALO, N. Y.
PUBLISHED BY JEWETT, THOMAS <t CO.
Commercial Advertiser Buildings.
1854.
Price $2.50 per annum, in advance.
				

